# Effects of chicory root powder on growth performance and histomorphometry of jejunum in broiler chicks 

**Published:** 2013

**Authors:** Homan Izadi, Javad Arshami, Abolghasem Golian, Mohammad Reza Raji

**Affiliations:** 1 *Department of Animal Sciences, Faculty of Agriculture,Ferdowsi University of Mashhad, Mashhad, Iran; *; 2 *Department of Pathobiology, Faculty of Veterinary Medicine, Ferdowsi University of Mashhad, Mashhad, Iran.*

**Keywords:** Broiler chicks, *Cichorium intybus*, Growth performance, Histomorphometry, Jejunum

## Abstract

In the present study, chicory root powder (CRP) as growth promoter at 1% and 3% levels was supplemented in broilers’ diet to investigate the growth performance and histomorphometry of jejunum. One hundred twenty, one-day-old male broilers were used in a completely randomized design (CRD) with 3 treatments and 4 replicates (10 chicks per replicate). At the end of each period (0-10, 11-24 and 0-24 days), feed intake (FI), weight gain (WG), and feed conversion ratio (FCR) were measured. At the end of experiment (day 24), one bird per replicate was sacrificed for breast weight (BW), drumstick weight (DW), and jejunum length (JL) as a percentage of body weight, and histomorphometry of villus. The FI increased by 3% CRP in the 1^st^ period (*p *< 0.01). The percentage of WG significantly increased at 1% during the 1^st^ period and, in the 2^nd^ and total periods, it increased only at 3% CRP (*p *< 0.05). The FCR decreased at 1% in the 1^st ^(*p* < 0.04) and, at 3% in the 2^nd^ (*p *< 0.01) and total periods (*p *< 0.05). The percentage of DW increased at 3% CRP (*p *< 0.05). The treatments increased the percentage of BW (*p *< 0.059) and, percentage of JL (*p *< 0.079) as well. The villus width and, crypt depth (CD) at 1% and 3% CRP and, villus surface at 3% reduced. The 3% CRP increased the villus length (VL) and villi number (*p* < 0.05) and, VL/CD (*p* < 0.01) and, villus surface area (*p *< 0.02). The percentage of leaf-like villi decreased in CRP treatments (*p *< 0.05). The number of goblet cells increased in CRP treatments (*p *< 0.01). In conclusion, chicory root powder can improve growth performance in broilers by enhancing food digestion and absorption through modification of jejunum histomorphometry.

## Introduction

Nowadays, poultry receive various supplementations such as antibiotics, growth promoters, vitamins, minerals, and even phytogenic plants to improve their performance and immunity. Using antibiotics as food additives for long periods in poultry diets can lead to antibiotic resistance and high residue levels in animal products such as meat and egg.^1^^,^^2^ Among the food additives, medicinal plants have drawn more attention these days due to their historical background and their prophylactic and growth promoter effects. Thus, the use of medicinal plants and probiotics in poultry diets for animal production and health has become more popular worldwide as an alternative to antibiotics.^3^

One of these plants is chicory (*Cichorium intybus*, Asteraceae) known as a promoter for immune system and growth in ancient nations such as Iran. The genus of chicory comprises about 14 species of herbaceous plants used in indigenous medicines.^4^ Chicory typically contains inulin (68%), sucrose (14%), cellulose (5%), protein (6%), ash (4%), and other compounds (3%), including esculin, coumarins, flavonoids, and vitamins in dry matter.^5^^,^^6^ The tuberous root of this plant contains a number of medicinally important compounds, including inulin, bitter sesquiterpene lactones, coumarins, flavonoids, and vitamins.^7^ Inulin, is a chain of fructans with non-soluble protein (NSP) which has minimal side effects, and is a good source of energy in an animal’s diet.^8^ Inulin regulates appetite and lipid-to-glucose metabolism with promising effects on body weight and fat mass development.^9^ Inulin-type fructans have been recognized as an interesting dietary ﬁbers that improve intestinal functions through their probiotic properties.^10^^,^^11^

The main industrial source of inulin and oligofructose is fresh chicory root.^12^ Chicory contributes to animal well-being in various ways. A promising effect of feeding inulin and oligofructose is decreasing the pH by increasing the absorption of short chain fatty acids. This effect could possibly be related to the thickening of the small intestine walls.^13^ Some studies suggest that intake of inulin and oligofructose enhances gastrointestinal absorption of minerals such as calcium, magnesium, and iron.^14^ This is related to protection against mineral deﬁciencies, and in the case of calcium, the prevention of osteoporosis.^15^ Addition of chicory root to diet improves the growth performance, egg production, and the length of small intestine in poultry.^16^^-^^18^ According to Yusrizal and Chen, the gradually fermented inulin significantly reduced serum cholesterol levels and fat tissue deposition in broilers.^16^ Other researchers reported that feeding inulin or oligo-fructose decreased circulating cholesterol and triglyceride levels.^19^ The aim of this study was to determine the effects of chicory root powder at 1% and 3% levels on growth performance and histomorphometry of jejunum in broiler chicks.

## Materials and Methods

**Birds and treatments. **The chicory roots were collected in autumn from (Aladagh mountain in Bojnourd, at 300 meter height), Iran. The chicory roots were dried in the oven at 50 ˚C for 48 hr, and then powdered. One hundred twenty, one-day-old male broiler chicks (Ross 308) were randomly divided into 12 groups (10 birds per group) with four replicates per treatment for a total of three different treatments. The dietary treatments were: I) basal diet as control, II) basal diet plus 1% chicory root powder (CRP) (10 kg per ton of diet), and III) basal diet plus 3% CRP (30 kg per ton of diet). The chicks received the starter diet from day 0-10 and the grower diet from day 11-24 of the study. The basal diet was formulated using corn and soybean meal according to the Ross Broiler Nutrient Specifications manual to 2.0-2.5 kg live weight (Table 1). The feed intake (FI), weight gain (WG) and feed conversion ratio (FCR) were calculated during the 1^st^ (0-10 day), 2^nd^ (11-24 day), and total (0-24 day) periods. On the last day of study (day 24), one bird per replicate (4 birds per treatment) was sacrificed to assess breast weight (BW), drumstick weight (DW), and jejunum length (JL), and the mean value was calculated as a percentage of body weight. The jejunum tissue of the birds was used for histological study. 

**Tissue sampling and preparation** ***. ***For histological study of villi, 2 cm tissue samples were taken from jejunum.^20^ The segments were flushed several times by 0.9% NaCl and fixed in fresh formaldehyde buffer (10%) for 48 hr.^21^ The tissue dehydration was carried out in graded alcohol (50%, 70%, 80%, 90%, 95%) and three times in absolute alcohol, and followed by embedding and fixation in paraffin. The tissue sections were harvested from 5 mm pieces with 5 µm thickness (three cross-sections from each sample) by microtome and fixed on slides and stained with Gill’s hematoxylin and eosin.^22^ Eight sections per segment in each replicate were utilized for histological study using an image analyser (Nikon Cosmozone 1S, Nikon Co., Ltd., Tokyo, Japan).

**Table 1:** Nutrient composition and calculated analysis (%) of the broiler basal diets.

**Diet**	**Starter** **(0 - 10 days)**		**Grower** **(11 - 24 days)**
***Ingredient***			
**Corn **	51.70		50.61
**Soybean Meal - 44%**	35.55		35.95
**Wheat Bran** *	4.00		4.00
**Sunflower Oil**	4.10		5.50
**Dical. Phos.**	1.64		1.42
**Limestone**	1.55		1.30
**Common Salt**	0.41		0.41
**Methionine **	0.22		0.22
**L-Lysine HCl**	0.33		0.08
**Vitamin-mineral premix** 1	0.50		0.50
***Calculated nutritive value***			
**ME, kcal kg** ^-1^	2924.00		3000.00
**CP (%)**	21.10		20.95
**Lysine (%)**	1.37		1.19
**Methionine + Cysteine (%)**	0.90		0.90
**Methionine (%)** **Calcium (%)**	0.54 0.99		0.54 0.86
**Available Phosphorous (%)**	0.47		0.43

* Wheat bran was replaced with 1% and 3% CRP in the treatments.

1 Vitamin-mineral premix provided the following per kg of diet: Vitamin A, 12500 IU; Vitamin D3, 2500 IU; Vitamin E, 18.75 mg; Vitamin K_3_, 2.65 mg; Vitamin B_1_, 2.00 mg; Riboflavin, 6.00 mg; Vitamin B_12_, 0.02 mg; Biotin, 0.03 mg; Folic acid, 1.25 mg; Pantothenic acid, 12.00 mg; Niacin, 50.00 mg; Copper, 8.00 mg; Zinc, 75.00 mg; Iron, 80.00 mg; Manganese, 100.00 mg; Selenium, 0.15 mg; Iodine, 0.35 mg; Salinomycin, 60.00 mg; Chlortetracycline, 0.10 g; Choline Chloride, 2.00 g; Ethoxyquin, 0.30 g.

**Characteristics of villus and crypt. **The images were analysed using stereological image software, Cast Image System (Version 2.3.1.3, Visiopharm, Horsholm, Denmark). The villus length (VL) was measured from the villus tip to the bottom (not including the intestinal crypt). A total number of 16 villi per section were measured in each replicate and four VL were averaged as the mean of villus length. Similarly, the crypt depths (CD) from the crypt-villus junction to the base of crypt, VL/CD, and villus width (VW) at mid-villus length were measured. The villus surface (VS) was calculated using the formula: VS = (2π) × (VW/2) × (VL).^23^ The villus number (VN) in 1000 µm^2^ was counted to calculate the villus surface area (VSA) by the formula: VSA = VS × VN. The goblet cell numbers (GCN) were counted from 5 villi per replicate in one randomly selected villus area (1000 µm^2^) and the mean value was calculated. The thickness of epithelia (ET), lamina propria (LPT), and muscle layers (MLT) were measured in the jejunum wall. 

**Fig. 1:**
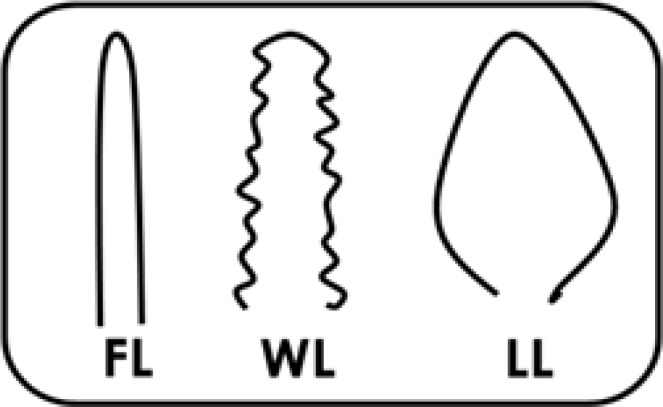
Schematic drawing of villus types: finger-like (FL), wave-like (WL), and leaf-like (LL).

**Fig. 2:**
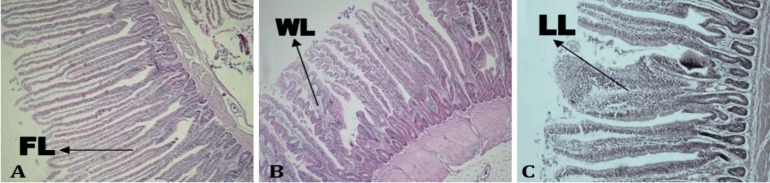
Histology of villus types; **A)** finger-like (FL), **B)** wave-like (WL), and **C)** leaf-like (LL) (H&E, 40×).

**Counting villus types. **The villus types are categorized into three shapes: 1) finger-like (FL) has a smooth surface, 2) wave-like (WL) has waves on the surface, and 3) leaf-like (LL) is wide in the middle (Fig. 1). The number of each type of villus in one selected area of jejunum (1000 µm^2^) was counted in each replicate and the mean was calculated as percentage (Fig. 2).

**Table 2:** Performance parameters in broilers fed chicory root powder during three periods (Mean ± SEM) (n=120).

	**Dietary treatments**				
**Parameters**		**Control**	**Chicory (1%)**	**Chicory (3%)**	***p *** **- value**
***First period (0-10 day)***					
**Feed intake (g)**		209.00 ± 7.00^b^	220.00 ± 7.00^ab^	232.00 ± 8.00^a^	0.009
**Weight gain (g)**		122.00 ± 5.00^b^	139.00 ± 4.00^ a^	141.00 ± 11.00^a^	0.011
**Feed conversion ratio**		1.71 ± 0.02^a^	1.53 ± 0.03^b^	1.59 ± 0.08^a^	0.040
***Second period (11-24 day)***					
**Feed intake (g)**		1039.00 ±75.00^a^	1076.00 ± 35.00^a^	1166.00 ± 119.00^a^	0.145
**Weight gain (g)**		603.00 ± 47.00^b^	672.00 ± 42.00^ab^	769.00 ± 87.00^a^	0.014
**Feed conversion ratio**		1.62 ± 0.01^a^	1.60 ± 0.05^a^	1.51 ± 0.02^b^	0.008
***Total period (0-24 day)***					
**Feed intake (g)**		1248.00 ± 82.00^a^	1296.00 ± 28.00^a^	1399.00 ± 125.00^a^	0.098
**Weight gain (g)**		725.00 ± 51.00^b^	812.00 ± 45.00^ab^	910.00 ± 95.00^a^	0.012
**Feed conversion ratio**		1.68 ± 0.10^a^	1.59 ± 0.05^ab^	1.53 ± 0.02^b^	0.044

ab Different letters in each row indicate significant differences (*p* < 0.05).

**Statistical analysis. **The experiment was carried out in a complete randomized design and the data were subjected to one-way analysis of variance (ANOVA) according to GLM procedure of SAS (Version 6.03, SAS Institute Inc., Cary, NC, USA). Differences between treatments were determined using the Duncan’s multiple range test and reported as means ± SEM.

## Results

**Growth performance** ***. ***The performance including feed intake (FI), weight gain (WG), and feed conversion ratio (FCR) are presented in Table 2. During the 1^st^ period, FI increased (*p* < 0.01) at 3% CRP, WG increased by both treatments (*p* < 0.01), and FCR reduced at 1% CRP (*p* < 0.04). In the 2^nd^ period, WG increased and FCR decreased at 3% CRP (*p* < 0.01). During the total period, 3% CRP increased WG (*p* < 0.01) and decreased FCR (*p* < 0.04). The overall results indicated that 1% and 3% CRP treatments improved growth performance in broilers during 24 days of study.

**Drumstick and breast weights and jejunum length** ***. ***The weights of drumstick (DW) and breast (BW), and the length of jejunum (JL) in broilers fed 1% or 3% CRP are presented in Table 3. The 3% CRP increased % of DW (*p* < 0.05). The percentage of BW and JL showed an increasing trend in treatments compared to those of control group (*p* < 0.059) and (*p* < 0.079), respectively. The results indicated that 3% CRP increased DW in broilers (*p*< 0.05).

**Villus parameters and crypt depth** ***. ***The results of morphological parameters of jejunum including villus width (VW), villus length (VL), crypt depth (CD), VL/CD, villus surface (VS), villus number (VN), and villus surface area (VSA) are displayed in Table 4. The 3% CRP increased VL and VN (*p* < 0.05) and, VL/CD (*p* < 0.01) and, VSA (*p* < 0.05). Both treatments decreased VW and CD, and VS. The morphological measurements revealed that 3% CRP increased VL, VL/CD, and VN which resulted in higher VSA and improvement of absorption and performance in broilers.

**Villus types** **and jejunum layers** ***. ***The results of morphological features of villus and jejunum layers including wave-like (WL), finger-like (FL) and leaf-like (LL) villi, epithelial thickness (ET), lamina propria thickness (LPT), muscle layer thickness (MLT), and goblet cell numbers (GCN) are presented in Table 5. The percentage of WL and FL villi increased by both treatments compared to those of control group. However, the LL villi decreased at 1% and 3% CRP in jejunum (*p* < 0.05). Both treatments decreased ET and MLT, and increased LPT compared to those of control group. The 1% and 3% CRP increased GCN in villi (*p* < 0.01). The results suggested that CRP treatments may improve jejunal absorption through morphological changes of villi and increasing GCN and LPT in jejunum.

**Table 3:** Mean ± SEM of body parts (%) of broilers fed chicory root powder at the end of the study (n=120).

**Parameters **	**Dietary treatments**			***p*** **-value**
	**Control**	**Chicory (1%)**	**Chicory (3%)**	
**Drumstick weight **15.93 ± 0.80^b^		17.09 ± 0.87^ab^	18.06 ± 0.67^a^	0.044
**Breast weight ** 16.14 ± 1.50^a^		18.17 ± 0.37^a^	18.48 ± 0.70^a^	0.059
**Jejunum length ** 0.39 ± 0.01^a^		0.40 ± 0.01^a^	0.42 ± 0.01^a^	0.079

ab Different letters in each row indicate significant differences (*p* < 0.05).

**Table 4:** Villus parameters of jejunum in broilers fed chicory root powder at the end of the study (Mean ± SEM).

**Parameters**	**Dietary treatments**			***p *** **- value**
	**Control**	**Chicory (1%)**	**Chicory (3%)**	
**Villus length (µm)**	912.00 ± 174.00^b^	1081.50 ± 137.00^ab^	1340.60 ± 1.10^a^	0.041
**Villus width (µm)**	151.50 ± 2.00^a^	130.00 ± 0.70^a^	123.20 ± 1.30^a^	0.094
**Crypt depth (µm)**	242.20 ± 4.40^a^	233.30 ± 1.00^a^	233.10 ± 2.10^a^	0.915
**Villus length/Crypt depth**	4.11 ± 0.20^b^	4.63 ± 0.40^ab^	5.75 ± 0.60^a^	0.010
**Villus surface (mm** ^2^ **)**	46.00 ± 13.00^a^	46.00 ± 4.00^a^	59.00 ± 10.00^a^	0.305
**Villus number**	4.50 ± 0.04^b^	5.05 ± 0.58^ab^	5.97 ± 0.73^a^	0.043
**Villus surface area (mm** ^2^ **)**	216.00 ± 48.00^b^	253.00 ± 32.00^ab^	373.00 ± 59.00^a^	0.019

ab Different letters in each row indicate significant differences (*p* < 0.05).

**Table 5:** Villus types and jejunum layers in broilers fed chicory root powder at the end of the study (Mean ± SEM).

**Parameters**	**Dietary treatments**			***p *** **- value**
***Villus types***	**Control**	**Chicory (1%)**	**Chicory (3%)**	
**Wave-like (%)**	28.67 ± 18.50^a^	43.33 ± 10.20^a^	33.67 ± 21.10^a^	0.600
**Finger-like (%) **	32.67 ± 11.00^a^	51.67 ± 17.60^a^	59.03 ± 19.30^a^	0.208
**Leaf-like (%) **	38.66 ±7.20^a^	5.00 ± 8.60^b^	7.30 ± 12.70^b^	0.011
***Jejunum layers***				
**Epithelial thickness (µm)**	4.09 ± 0.20^a^	3.76 ± 0.60^a^	3.82 ± 0.50^a^	0.689
**Lamina propria thickness (µm)**	112.83 ± 21.00^a^	133.58 ± 12.00^a^	144.83 ± 21.00^a^	0.190
**Muscle layer thickness (µm)**	4.82 ± 0.52^a^	4.55 ± 0.53^a^	4.27 ± 0.85^a^	0.692
**Goblet cell numbers**	8.76 ± 1.10^b^	11.44 ± 0.40^a^	12.52 ± 1.30^a^	0.002

ab Different letters in each row indicate significant differences (*p* < 0.05).

## Discussion

Recent researches focus on chicory that has major fibre components and potential probiotic function due to inulin-type fructans and oligofructose.^5^^,^^6^^,^^24^ Addition of 1% and 3% CRP increased growth performance of broilers in comparison with control group. Moreover, feeding chicory to broilers led to increases in percentage of DW and percentage of BW. Also, chicks fed with 3% CRP had higher GW than those received 1% CRP in their diet for 24 days. Our results are in agreement with Yusrizal and Chen who reported that birds received 1% oligofructose were heavier, especially female broilers (10%).^16^ However, feeding fresh chicory to young rabbits slightly inhibited feed intake, and weight gain during the pre-weaning period.^25^ Whereas, another study showed adding fructo-oligosaccharide to the basal diet (0.37%) of broilers had positive effects on performance.^26^ Addition of 3% CRP decreased FCR during the total period in broilers (*p* < 0.05). Similarly, Ammerman *et al.*, and Yusrizal and Chen reported lower FCR in parallel with better growth.^16^^,^^26^ Although, a previous report showed that feeding moderate levels of inulin (5-10%) did not speed up growth rate.^27^ Earlier work indicated unaltered weight gains in rats fed with different kinds and levels of NSP compared to control group.^5^ According to Montagne *et al.*, a diet with high content of fibre will result in dilution of energy.^2^ This implies that chicory should be incorporated as an additive rather than a food.^25^

The results of our study demonstrated that feeding 1% and 3% CRP to broilers improved growth performance by enhancing absorption through significant increase of VL, VL/CD, VN and VSA and decreased of VW, CD and VS in villi.

Therefore, we can conclude that enhancements of length, number and surface area of villi are paralleled with an increased digestive and absorptive capacity of the jejunum. Basically, villi develop rapidly and continuously in response to lumen conditions reflecting the dynamic inner environment of the animals’ gut. Longer villi result in definite gain of intestinal surface area. Notably, a few morphological studies showed a significant enlargement of villus length or crypt depth of the small intestine from monogasteric animals after feeding chicory NSP and/or inulin fructans.^18^^,^^28^^,^^29^ In our study, the percentage of JL increased by CRP treatments (*p* < 0.079), whereas Iji *et al*. reported no significant differences between broilers fed with commercial NSP diets and control group in the morphometry of the intestinal mucosa.^30^ The thickness of jejunal muscularis external of rats given chicory and pectin in their diets was not affected.^5^ The results of this study showed a gradual increase in LPT with decreasing in MLT and ET in broilers fed with CRP during 24 days with no significant differences between treatments. Also, the increased LPT affected on growth and development of villi in jejunum. Moreover, increasing the GCN by treatments was associated with more pronounced absorptive capacity in jejunum (*p* < 0.01). These findings demonstrated that CRP at 1% and 3% levels promoted digestion and absorption through the histomorphological changes of villi and jejunum parameters. In fact, CRP content caused villi to grow faster as a result of LPT development which leads to harvesting more energy and nutrients. The morphometry of jejunum was also affected by the CRP treatments (*p* < 0.05). 

In conclusion, supplementing CRP in diet enhanced growth performance in broilers. Improving digestion and absorption during the starter period is detrimental to the health of broilers. Adding CRP at 3% provided sufficient improvement through morphological changes of villi in the jejunum to maintain growth and development. The long term effects of CRP on health status and production performance in broilers, pullets, and laying hens are yet to be quantified.
